# On Slip Detection for Quadruped Robots

**DOI:** 10.3390/s22082967

**Published:** 2022-04-13

**Authors:** Ylenia Nisticò, Shamel Fahmi, Lucia Pallottino, Claudio Semini, Geoff Fink

**Affiliations:** 1 Dynamic Legged Systems (DLS) Lab, Istituto Italiano di Tecnologia (IIT), Via S. Quirico 19D, 16163 Genova, Italy; ylenia.nistico@iit.it (Y.N.); claudio.semini@iit.it (C.S.); 2 Università di Pisa, Scuola di Ingegneria, Via Diotisalvi 2, 56122 Pisa, Italy; lucia.pallottino@unipi.it; 3 Biomimetic Robotics Lab, Massachussetts Institute of Technology (MIT), 77 Massachusetts Ave., Cambridge, MA 02139, USA; sfahmi@mit.edu; 4 Thompson Rivers University, Department of Engineering, 835 University Dr., Kamloops, BC V2C 0C8, Canada

**Keywords:** legged robots, perception, slip detection

## Abstract

Legged robots are meant to autonomously navigate unstructured environments for applications like search and rescue, inspection, or maintenance. In autonomous navigation, a close relationship between locomotion and perception is crucial; the robot has to perceive the environment and detect any change in order to autonomously make decisions based on what it perceived. One main challenge in autonomous navigation for legged robots is locomotion over unstructured terrains. In particular, when the ground is slippery, common control techniques and state estimation algorithms may not be effective, because the ground is commonly assumed to be non-slippery. This paper addresses the problem of slip detection, a first fundamental step to implement appropriate control strategies and perform dynamic whole-body locomotion. We propose a slip detection approach, which is independent of the gait type and the estimation of the position and velocity of the robot in an inertial frame, that is usually prone to drift problems. To the best of our knowledge, this is the first approach of a quadruped robot slip detector that can detect more than one foot slippage at the same time, relying on the estimation of measurements expressed in a non-inertial frame. We validate the approach on the 90 kg Hydraulically actuated Quadruped robot (HyQ) from the Istituto Italiano di Tecnologia (IIT), and we compare it against a state-of-the-art slip detection algorithm.

## 1. Introduction

We live in an era of rovers on Mars [1], drones surveying Earth (and Mars [2]), and self-driving cars. Similarly, legged robots have gained increasing popularity over the past few decades. They have the potential to operate in unstructured and dangerous environments, and to traverse difficult terrain where existing vehicles cannot go.

Legged robotics has seen significant progress in recent years. Robots have acquired amazing capabilities and some of them have reached a point where they can actually leave lab environments and carry out tasks in real-world scenarios. For example, the humanoid robot Atlas of Boston Dynamics [3], has recently demonstrated impressive athletic behaviors by performing parkour [4]. On the other hand, typical biped robots lack locomotion stability in unstructured environments, whereas quadruped robots have good mobility and stability. Among quadruped robots, the Hydraulically actuated Quadruped robots HyQ and HyQReal [5,6] were designed at the IIT (Genoa) to study highly dynamic motions (e.g., running, hopping, jumping), and to navigate over rough terrain. HyQReal demonstrated a strong heavy-duty ability by pulling a small airplane in an experiment [7]. The research group of Swiss Federal Institute of Technology (Zurich) and Anybotics AG developed the quadruped robot ANYmal [8], for autonomous operation in challenging environments [9]. Massachusetts Institute of Technology (MIT) introduced the electrically actuated quadruped robot MIT Cheetah [10] and later MIT Cheetah 3 [11] to study high-speed quadrupedal locomotion. One of the most popular quadruped robots is Spot [12], constructed by Boston Dynamics. Spot is a versatile quadruped robot, suitable for many applications. It demonstrated great ability in navigating several kinds of terrain while sensing the environment. Unitree Robotics developed Aliengo [13] and other electric quadrupeds that demonstrated excellent athletic performance, such as fast running, jumping, and climbing. A recent survey paper [14] presents the results obtained with some of the best-known quadruped robots, including Spot, ANYmal, and HyQ.

More recently, the researchers of the Robotic Systems Lab, ETH Zurich, Switzerland, presented a controller tuned by reinforcement learning [15]. Their controller was based on a neural network that operates proprioceptive signals. Despite having performed simulations and training on rigid and not very uncertain terrain, thanks to reinforcement learning, once the robot (ANYmal) was taken to real, uncertain, and rich-in-vegetation terrain, it was able to complete the required tasks. It also demonstrated a certain robustness to slippage, despite not having implemented a real slip detection.

It is clear that the improvements achieved on a hardware and control level go hand in hand with the perceptive capabilities. As part of this, state estimation adopts a central role since estimated quantities are prerequisites for other tasks such as balance control, trajectory planning, or target tracking [16].

To move out of research labs into the real-world, legged robots are expected to traverse terrains that are usually dynamic, unexplored, and unknown. The core problem is that the terrains that the robots have to traverse introduce a large amount of uncertainty. The robot has to be *terrain-aware*, that is to say, able to perceive and understand the surrounding terrain, and to take decisions based on that. Proprioceptive Terrain Aware Locomotion (PTAL) relies on internal robot measurements, using information acquired by proprioceptive sensors. PTAL strategies are effective in many scenarios where it is difficult to obtain visual feedback (e.g., foggy areas). Common PTAL strategies are used to localize and detect contacts. Some techniques rely on the joint position, velocity, and/or torque measurements to detect foot-ground contacts, or localize other possible contacts with the environment [17,18,19]. Furthermore, PTAL strategies are used to deduce the physical and geometrical properties of the terrain and adapt accordingly [20].

This work focuses on PTAL, applied at the level of state estimation, and investigates locomotion on slippery terrain, an open and challenging research problem in dynamic locomotion. The final goal is to develop a new algorithm for multi-leg slip detection during motion, making use of proprioceptive sensors.The development of recovery strategies once slippage is detected, is part of our future work.

### 1.1. Related Work on Slip Detection and Recovery

Some work dealt with slip detection and recovery by focusing on the design of the robot’s foot. For example, in [21] an anti-skid foot designed on a two-legged planar robot is introduced. The robot’s foot has two types of foot pads: a primary foot pad with rubber, and a secondary (complementary) foot pad with anchoring spines. The foot switches from the first to the second when the primary foot pad slips.

A method to prevent a slip event is described in [22]. The authors presented a *MEMS* slip sensor that can be attached to a foot of a legged robot to measure the slipperiness at the time of the collision between the foot and the ground, in order to prevent the slippage in static and dynamic situations.

A study on detection of “absence of slip in robot hand and feet” was made in [23], by collecting data from independent 3-axis *MEMS* accelerometers connected to pads, that could be used both for gripping configuration and for foot configuration. Based on the fact that, in absence of slip, the sensor signals are highly correlated, the authors developed an algorithm of non-slip detection, able to make a fast non-slip/slip decision. The results showed that the robustness of the algorithm is lower in the case of foot configuration, probably due to a greater loss of the signals acquired during the different contacts.

The abovementioned results demonstrate that sensor-based slip detection approaches are of limited applicability to legged robots, because they need a force/torque sensor to be attached to the foot tip. Due to the repetitive impacts with the ground, in the long run, this can result in damage to the sensor. Furthermore, during locomotion, the touchdown event can create discontinuities in the force signal and jeopardize the detection. Conversely, a detection strategy based on kinematics is preferable in the context of legged robots where ground impacts are the order of the day.

One of the prior works on slip detection and recovery is from [24]. Here the authors proposed to change the robot’s gait parameters (gait frequency and stride length) when approaching slippery surfaces (*long-term strategy*), and to instantaneously add a force to have the Ground Reaction Force (GRF) back in the friction cone (*short-term strategy*). This approach is based on the assumption that the normal force is always correctly estimated. The method was tested on the quadruped robot *TITAN-VIII*.

For the bipedal robot *HRP-2*, a slip observer detecting skids occurring at walking on slippery floor was formulated [25]. To realize balance control, a reactive strategy was achieved by regulating the desired footholds to compensate for the torso rotation caused by the slippage.

The implementation of an Invariant Extended Kalman Filter (InEKF) that fuses inertial, velocity measurements from leg kinematics and from a tracking camera, for legged robots operating in slippery environments, is proposed in [26]. The authors experimentally validate the proposed method on a Cassie bipedal robot walking over slippery terrain.

In [16] a state estimation approach based on kinematic velocity measurements at the ground contacts is presented. The obtained information is fused with measurements from an onboard IMU by means of an Unscented Kalman Filter (UKF), implemented on the legged robot StarlETH. This filter estimates roll and pitch angles as well as the velocities of the robot, it is robust to a certain amount of foot slippage, and enables dynamic locomotion over uneven and unstable terrain.

In [27] the authors introduce a methodology for slip detection and estimation of the friction parameters, plus a recovery strategy, which exploits the capabilities of a whole-body controller, implemented for HyQ locomotion, which optimizes for the GRFs. The estimation makes use only of proprioceptive sensors. The detection approach is fully described in Section 3 since it is used as a baseline for the novel algorithm presented in this paper.

In [28] a probabilistic approach for contact and slip estimation, based on a Hidden Markov Model is developed and tested on ANYmal walking on frozen ground. A slip recovery approach relied on invasive impedance control and friction modulation. The authors performed field tests on frozen ground, verifying that the presented approach could successfully stabilize ANYmal.

### 1.2. Contributions

This paper focuses on proposing a new approach for slip detection. This is the first essential step to implement a robust controller that allows the robot to navigate on slippery ground. Once the robot detects slippage, it can trigger a reflex action and adapt its motion trajectories to stop slipping.

Previous works on slip detection rely on estimating the robot states in an inertial frame (*world frame*), in particular the foot velocity being constant in the world frame. These measurements can be influenced by errors in the state estimation, which can result in false positives in slip detection. Moreover, some of them rely on the integration of the acceleration measured by the IMU. Integrating acceleration is prone to drift and can cause divergence issues. Our method overcomes the challenges of prior work on slip detection by not relying on the robot state estimates in an inertial frame. Instead, our method relies on estimating the velocity and the position of each foot with respect to the body of the robot (*base frame*), in order to avoid drift and divergence issues that arise when using the world frame.

Furthermore, we propose a kinematic-based algorithm that is independent of the type of gait. Our approach can independently detect the slippage of one or more legs at the same time. This allows us to detect slippage if the robot is walking with a trot gait (two legs are swinging at the same time), or with a crawling gait (only one leg is swinging).

Additionally, we validated the proposed approach on the 90 kg Hydraulically actuated Quadruped (HyQ) robot, and we compared it against a state-of-the-art slip detection algorithm [27].

### 1.3. Outline

The rest of this paper is structured as follows: Section 2 gives a system overview of HyQ and the locomotion framework used in this work. Section 3 gives a recap of the work presented in [27], as well as the proposed algorithm of slip detection. Section 4 shows the simulation results. Section 5 shows the results obtained in experiments, and finally Section 6 is dedicated to conclusions and future works.

## 2. Modelling and Sensing

### 2.1. Robot Overview

**Figure 1:**
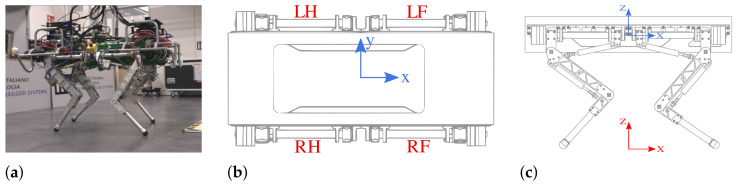
(**a**) HyQ robot, (**b**,**c**): location of robot base frame (blue) and world frame (red). LF, RF, LH, and RH are the Left-Front, Right-Front, Left-Hind, and Right-Hind legs, respectively.

HyQ (Figure 1a) is a fully torque-controlled hydraulically actuated quadruped robot developed at the IIT. HyQ stands 1 m tall and weighs approximately 90 kg. HyQ consists of a torso and four identical legs, arranged in the forward/backward configuration, with the front and hind knees pointing to each other. It has 12 torque-controlled joints powered by hydraulic actuators. The actuated joint are: (i) Hip Abduction/Adduction (HAA), (ii) Hip Flexion/Extension (HFE), (iii) Knee Flexion/Extension (KFE). For more technical specification the reader can refer to [5].

We define the reference frames as shown in Figure 1b,c: the base frame B located at the geometric center of the trunk (robot torso) and the world frame W, an inertial frame coinciding with the base frame in the starting position, considering an offset along the *z*-axis of exactly the height of the robot.

### 2.2. Sensors Overview

HyQ is equipped with a six-axis IMU on the trunk, and every joint contains an encoder and torque sensor. The encoders are used to measure the joint position $q_{i}\in R$ and joint speed ${\dot{q}}_{i}\in R$. The torque sensors directly measure joint torque $\tau_{i}\in R$. The bias and noise of the measured values are assumed slowly time-varying, with zero mean and Gaussian distribution. HyQ is equipped with proprioceptive and exteroceptive sensors. For this work, the information is obtained exclusively from proprioceptive sensors data. A complete description of the sensors the robot is equipped with is reported in [29].

### 2.3. Dynamic Model

The dynamics of the robot is obtained starting from the assumption that all of the external forces are exerted on the point-like feet. When a foot is in the *swing* phase (no foot-ground contact) it is assumed that no external forces are exerted on it. When the contact occurs, the foot is in *stance* phase, and the ground exerts forces on it (GRFs). The dynamics model is given by:

(1) $$M\left(\bar{x}\right)\ddot{\bar{x}}+h\left(\bar{x},\dot{\bar{x}}\right)=\bar{\tau }+J^{T}F_{grf}$$

where $\bar{x}={\left[x^{T}\eta^{T}q^{T}\right]}^{T}\in R^{18}$ is the generalized robot state, $\dot{\bar{x}}\in R^{18}$ and $\ddot{\bar{x}}\in R^{18}$ are the corresponding generalized velocities and accelerations, $x\in R^{3}$ and $\eta \in R^{3}$ are the position and the attitude of the base, $q\in R^{12}$ is the vector of joint angles (3 DoF for each leg), $M\in R^{18\times 18}$ is the joint-space inertia matrix, $h\in R^{18}$ is the vector of Coriolis, centrifugal and gravity forces, $\hat{\tau }=\left[\underline{0}\;\tau \right]\in R^{18}$ where $\tau \in R^{12}$ is the vector of joint torques, and finally $F_{\mathrm{grf}}\in R^{12}$ is the vector of GRFs, while $J\in R^{18\times 12}$ is the floating base Jacobian.

In this paper, estimating the contact states of the feet (whether a foot is in contact with the ground or not) is essential. To estimate the contact states $\alpha \in R^{4}$, we first estimate the ground reaction forces as detailed in [30]. Then, the contact state $\alpha_{i}$ for every leg *i* is a boolean variable whose value is equal to 1 when the GRFs exceed a certain threshold value, and is equal to 0 otherwise. In detail, the contact state $\alpha_{i}$ is defined as:

(2) $$\alpha_{i}=\left\{\begin{array}{cc} 1 & F_{\mathrm{grf},i}>F_{\min}\\ 0 & otherwise \end{array}\right.$$

where $F_{\min}$ is the threshold value (50N in the experiments of Section 5), and $F_{\mathrm{grf},i}\in R^{3}$ is the GRF of the leg *i*.

### 2.4. Locomotion Framework

**Figure 2:**
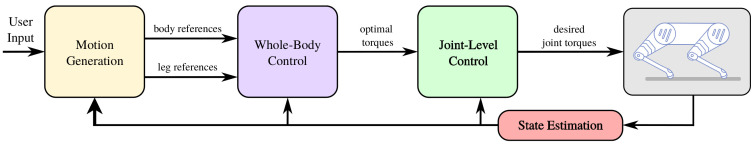
Overview of the locomotion framework.

The locomotion framework used in this work is based on the *Reactive Controller Framework* (RCF) described in [31]. The RCF comprises four main blocks. The first block is *motion generation* which takes user-defined inputs and outputs body and leg references, and adjusts them with reflex strategies. The second block is *Whole-Body Control* (WBC) that tracks the references from the motion generation block and outputs optimal torques [32]. The third block is *joint-level control* that tracks the optimal torques from the WBC block and outputs the desired joint torques to send them to the robot. The fourth block is *state estimation* that estimates the robot (body, joint, and contact) states from the sensor measurement of the robot and sends them to the motion generation and the WBC blocks. An overview of the locomotion framework is shown in Figure 2.

HyQ has two main locomotion behaviors [33]: a dynamically stable trot and a crawl-gait. During the trotting the robot moves its legs in pairs. Trotting allows fast locomotion over regular terrain with varying inclinations. The crawl-gait is slow locomotion, more suitable for irregular terrains. The crawling pattern is LH to LF to RH to RF. For the simulation and experimental tests (Section 4 and Section 5) we used both these methods of locomotion.

## 3. Slip Detection Method

In this section, we briefly introduce a state-of-the-art algorithm [27], used as a baseline approach for comparison. Then, after explaining the drawbacks of the baseline approach, we explain our proposed approach.

### 3.1. Baseline Approach

The baseline approach [27] proposed two different strategies to detect slippages. These strategies were based on the robot kinematics, particularly at the velocity level. The first strategy detects the slippage of only one leg, and is based on estimating the stance feet velocities expressed in the body frame ${\dot{x}}_{f}^{b}$. The second strategy detects the slippage of two or more legs, and is based on estimating the stance feet velocities expressed in the world frame ${\dot{x}}_{f}^{w}$.

#### 3.1.1. One Leg Slip Detection

For the first strategy, the authors propose to compare the values of stance feet velocities in the body frame ${\dot{x}}_{f}^{b}$ and discriminate the outlier with appropriate statistical tools. At each control loop the median of the norms of the stance feet velocities is computed. A slipping leg has a velocity that deviates the most from the median beyond a certain threshold ϵ, that is experimentally tuned. Each leg has an associated flag, that is switched to *true* if a slip is detected.

#### 3.1.2. Multiple Leg Slip Detection

A more complicated situation is when two or more legs are slipping at the same time. In this case, it is hard to detect with the median approach which legs are slipping or in stance. Therefore, the authors proposed a second strategy, that is checking which of the feet velocities ${\dot{x}}_{f}$ are kinematically consistent with the base velocity ${\dot{x}}_{b}^{b}$. The most intuitive way is to verify that the Cartesian velocities of the stance feet ${\dot{x}}_{f}^{w}$ are all *zero* in an inertial frame W. ${\dot{x}}_{f}^{w}$ can be written as follows:

(3) $$0≃{\dot{x}}_{f}^{w}={\dot{x}}_{b}^{w}+R_{b}^{w}\left({\dot{x}}_{f}^{b}+\omega \times x_{f}^{b}\right)$$

where $R_{b}^{w}\in \mathrm{SO}\left(3\right)$ is the rotation matrix representing the orientation of the robot base (i.e., from frame B to frame W). $x_{f}^{b}\in R^{3}$, ${\dot{x}}_{f}^{b}\in R^{3}$ are the position and velocity of the foot in B, respectively, ω is the angular velocity, estimated by an on-board IMU sensor, while ${\dot{x}}_{b}^{w}\in R^{3}$ is the base linear velocity in W. According to (3), computing ${\dot{x}}_{f}^{w}$ requires an estimation of the base linear velocity ${\dot{x}}_{b}^{w}$. For this purpose, a short-time integration of the base linear acceleration measured by the IMU accelerometers is executed.

#### 3.1.3. Drawbacks of the Baseline Method

The slip detection approaches described above have some downsides. They were tested only in simulation, using *crawl* as gait. The *one leg slip detection* method, cannot be applied to different gaits, e.g., *trotting*, where the legs have pairwise different velocities and the accuracy might deteriorate. On the other hand, the *multiple leg slip detection* method relies on velocities expressed in the *world frame*. ${\dot{x}}_{b}^{w}$ is influenced by errors in the state estimation, which can result in false positives in slip detection. Moreover, this approach relies on the integration of the acceleration measured by the IMU. As already explained in Section 1.2, integrating acceleration is prone to drift and it is fundamental to perform a *short-time* integration to avoid divergence issues. The authors did not perform experiments on a real robot, therefore it cannot be said with certainty that it is actually robust.

### 3.2. A Novel Approach for Slip Detection

The idea behind the proposed method is to overcome the aforementioned problems. We move to the idea of detecting the slippage using feet velocities expressed in B. An approach based on ${\dot{x}}_{f}^{b}={\left[{\dot{x}}_{f_{x}}^{b}{\dot{x}}_{f_{y}}^{b}{\dot{x}}_{f_{z}}^{b}\right]}^{T}\in R^{3}$ is more robust because it directly depends on direct sensor measurements (e.g., encoders), avoids drifts that usually happen in getting the base states in the world frame, or using numerical integration from IMU.

A slipping leg has the foot velocity deviating from the desired one. A measure of the deviation from the desired velocity can be given by $\Delta V={\left|\right|}_{d}{\dot{x}}_{f}^{b}-{\dot{x}}_{f}^{b}\left|\right|$, the norm of the difference between the desired and actual foot velocities in the body frame B.

**Figure 3:**
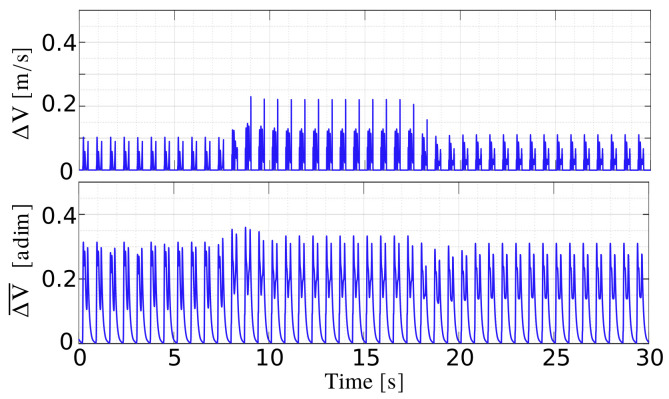
ΔV (**top**) and $\bar{\Delta V}$ (**bottom**) in a simple trotting task with variable feed rate (no slippage). The higher peaks in the top-figure correspond to a higher linear velocity along the *x*-axis.

We can thus detect slippage, when ΔV exceeds a certain limit, e.g., a threshold ϵ, during the stance phase. However, calculating the norm is not a reliable tool for detecting slippage. The difference between the desired and actual velocity increases along the prevalent direction of motion because of a greater tracking error. Furthermore, if in general we want to change the velocity of the robot during its motion, we need to adjust ϵ accordingly. So to ensure that the direction and velocity of motion do not affect ΔV, we introduce a weight to scale each component of the vector ${}_{d}{\dot{x}}_{f}^{b}-{\dot{x}}_{f}^{b}$. The weight is ${}_{d}{\dot{x}}_{f_{i}}^{b}$, the desired foot velocity in B for each component. In this way we minimize the impact of a possible greater tracking error in one of the three directions, and we ensure to have a $\bar{\Delta V}$ that remains similar during the motion, even if we change the desired body velocity. This is evident in Figure 3: on the top of the figure, it is shown how ΔV changes when the velocity of the robot is modified, during a trotting task with a variable feed rate (the velocity increases in the interval [10–20] s, along the *x*-direction). On the bottom, it is shown that $\bar{\Delta V}$ keeps the same trend for the duration of the previous task.

For numerical reasons, to ensure that the denominator is always non-zero, we divide by ${|}_{d}{\dot{x}}_{f_{i}}^{b}|$ and we add a margin *m*, whose value is experimentally tuned. When slippage occurs, the value of $\bar{\Delta V}$ increases. So we impose an upper limit to $\bar{\Delta V}$, beyond which a further increase is considered a slippage. In the end, we have:

(4) $$\bar{\Delta V}=\sqrt{\sum_{i=x,y,z}{\left(\frac{{}_{d}{\dot{x}}_{f_{i}}^{b}-{\dot{x}}_{f_{i}}^{b}}{{|}_{d}{\dot{x}}_{f_{i}}^{b}|+m}\right)}^{2}}\;>\;\epsilon_{v}$$

where $\epsilon_{v}$ is a *conditioned* threshold because its value depends on the phase of the foot motion. During the *swing phase*$\epsilon_{v}$ is set to *∞* because a swing-leg cannot slip. During the *stance phase*$\epsilon_{v}$ has a constant value, for each leg. This value is chosen as the percentile on $\bar{\Delta V}$. The percentage *p* is tuned in order to detect the higher peaks of $\bar{\Delta V}$.

(5) $$\epsilon_{v}=\left\{\begin{array}{cc} \infty & swing\;phase\\ percentile(\bar{\Delta V},p) & stance\;phase \end{array}\right.$$

**Figure 4:**
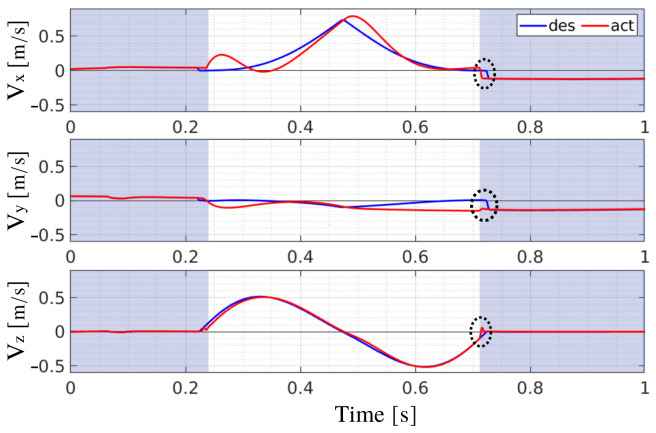
Desired (blue line) and actual (red line) foot velocity in a simulation task of crawling (no slippage). Velocity $v=[v_{x}\;v_{y}\;v_{z}]$ is expressed wrt B. The shaded areas indicate that the foot is in stance.

In case of slippage, another important question is: *How far did we slip?* A quantitative measure of the slipping length can be derived from the measurement of the foot *position*. Indeed, during slippage, the foot position changes by deviating from the desired one. This deviation can be quantified by calculating $\Delta P=\left|{\left|\right|}_{d}x_{f_{i}}^{b}\left|\right|-\left|\right|x_{f_{i}}^{b}\left|\right|\right|$. ΔP is important for another reason: at the beginning of a foot-ground contact, there is a short time interval in which the difference between desired and actual foot velocities instantaneously increases (Figure 4). This may be due to multiple causes: (i) an actual, but small and, perhaps, not important slippage, (ii) delay in control, and (iii) the current implementation of stance detection (Section 2.3) that leads to a small delay in detecting contact with the ground. The foot slippage (∼1–2 cm) in this time interval is considered negligible if compared to the size of the robot, and to make sure it is not detected we add a condition on ΔP:

(6) $$\Delta P=\left|{\left|\right|}_{d}x_{f_{i}}^{b}\left|\right|-\left|\right|x_{f_{i}}^{b}\left|\right|\right|>\epsilon_{p}$$

If ΔP remains below the threshold $\epsilon_{p}$, the slippage is considered acceptable. $\epsilon_{p}$ has a constant value, tuned experimentally.

Therefore, a slippage is detected when both $\bar{\Delta V}$ and ΔP exceed their respective thresholds. We introduce a flag $\beta_{i}\in \left[0,1\right]$ for each leg *i* whose value is 1 if there is a slip detection, 0 otherwise. Below the pseudo-code implementation of the proposed Algorithm 1.
**Algorithm 1:** detectSlippage(${\dot{x}}_{f_{i}}^{b}$,${}_{d}{\dot{x}}_{f_{i}}^{b}$,$x_{f_{i}}^{b}$,${}_{d}x_{f_{i}}^{b}$)1: $\bar{\Delta V}$←$scaled\;diff({\dot{x}}_{f_{i}}^{b}{,}_{d}{\dot{x}}_{f_{i}}^{b})$;  ▹${\dot{x}}_{f_{i}}^{b}$ and ${}_{d}{\dot{x}}_{f_{i}}^{b}$ are the *actual* and *desired* foot velocity in B
2: $\Delta P\leftarrow diff(x_{f_{i}}^{b}{,}_{d}x_{f_{i}}^{b})$;     ▹$x_{f_{i}}^{b}$ and ${}_{d}x_{f_{i}}^{b}$ are the *actual* and *desired* foot position in B3: **for** each stance leg *i*
**do**4:   $\beta_{i}$←$\left(\bar{\Delta V}>\epsilon_{v}\right)\;\&\;\left(\Delta P>\epsilon_{p}\right)$;     ▹$\epsilon_{v}$ and $\epsilon_{p}$ are the thresholds for $\bar{\Delta V}$ and ΔP5: **end**


## 4. Simulation Results

The robot kinematics and dynamics functions are implemented in C++, as well as the proposed algorithm. We used the software simulator Gazebo to test and refine our approach. To integrate the work with the other elements of the robot we built the proper ROS structure. Then all the data collected from the simulations were analyzed in Matlab in order to improve the phase of tuning. Simulation (and experimental) results are shown in the attached video.

We performed several simulations to test the proposed and the baseline approach. Then we compared the results in order to underline the strengths of our method.

### 4.1. Crawling onto Patches of Ice

**Figure 5:**
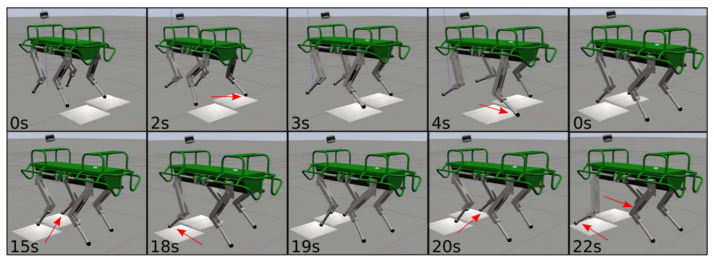
Simulation of the HyQ robot traversing slippery terrain patches with a crawl gait (low friction patches illustrated in white). The image sequence starts from the top left to right and continues at the bottom left. The red arrows indicate slipping feet.

A transition from walking on flat terrain (μ=0.8) to ice slabs (μ=0.08) is a good template to demonstrate the effectiveness of the algorithm. Figure 5 shows the sequence of a movement: the robot starts walking from non slippery terrain and then moves to ice-sheets. During the motion, all the legs are slipping. For this simulation test we used the following parameters: $\epsilon_{v}=percentile\left(\bar{\Delta V},99\%\right)$, $\epsilon_{p}=0.03$, m=0.3.

**Figure 6:**
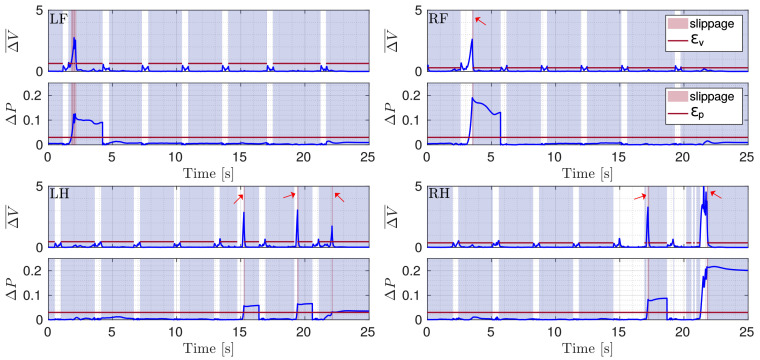
Plots of $\bar{\Delta V}$ and ΔP (blue) with respective thresholds (red line) illustrated for the four legs (LF, RF, LH, RH) during a crawl gait. The gray shaded area shows stance phases and the red shaded area marks slippage. The little red arrows indicate short slip events.

In Figure 6 the shapes of $\bar{\Delta V}$ and ΔP are shown. The shaded areas represent the periods when a foot is in contact with the ground. The light red stripes indicate the slippage. Legs slip in the order LH-RF-LH-RH. The change in velocity of a slipping leg affects the $\bar{\Delta V}$ shapes of the other legs. This is because the velocities are expressed in B. When a foot is slipping, the base-position changes for some instants, and then each foot velocity changes (top figures in each subplot). Adding the further constraint on ΔP (6) prevents this from affecting the correct detection for the other legs.

As it is possible to see in the attached video, in this test the slippage occurs one leg at a time. So, for the comparison we implemented the baseline strategy described in Section 3.1 to detect the slippage of one leg. As threshold for the baseline approach we chose $\epsilon_{v_{BL}}=0.04$.

**Figure 7:**
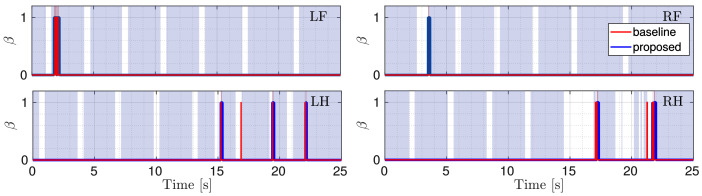
Comparison between the flags. The red one is obtained with the baseline approach, the blue one with the proposed approach. The gray shaded area shows stance phases and the red shaded area indicates the ground truth.

Figure 7 shows the flags obtained with the proposed approach (blue lines) and the baseline (red lines). The shaded red areas indicate when the robot is truly slipping, i.e., the ground truth. We manually obtained this ground truth from simulation where we observed the periods when the robot was slipping. From Figure 7 it is clear that our approach is more reliable in detection: it is faster and the flag remains 1 for the entire duration of the slippage.

### 4.2. Trotting onto Patches of Ice

**Figure 8:**
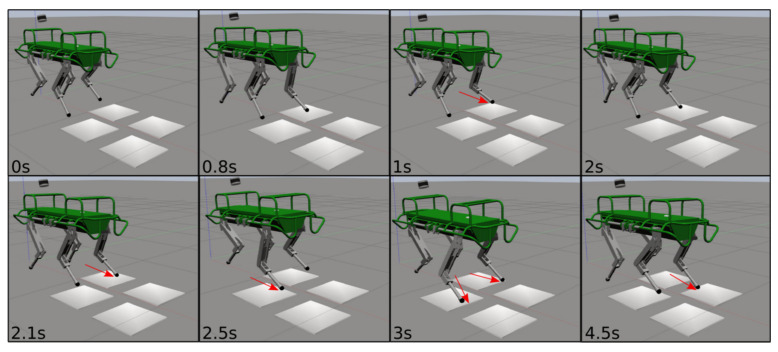
Simulation of the HyQ robot traversing slippery terrain patches with a trot gait (low friction patches illustrated in white). The image sequence starts from the top left to right and continues at the bottom left. The red arrows indicate slipping feet.

To demonstrate that the proposed method can be used with different gaits, we tested it in simulation with the robot trotting on a terrain consisting of four low friction patches (μ=0.08), as shown in Figure 8. We used the following parameters: $\epsilon_{v}\;=\;percentile\left(\bar{\Delta V},95\%\right)$, $\epsilon_{p}=0.03$, m=0.3. For the comparison we implemented the baseline strategy described in Section 3.1 to detect the slippage of multiple legs slipping at the same time. For the baseline we set the threshold $\epsilon_{v_{BL}}=1$.

**Figure 9:**
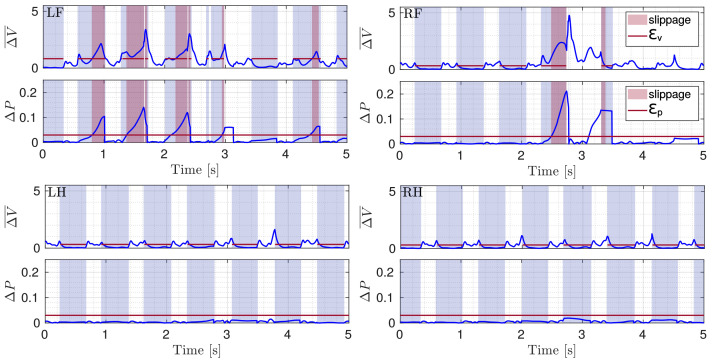
Plots of $\bar{\Delta V}$ and ΔP (blue) with respective thresholds (red line) illustrated for the four legs (LF, RF, LH, RH) during a trot gait. The gray shaded area shows stance phases and the red shaded area marks slippage.

**Figure 10:**
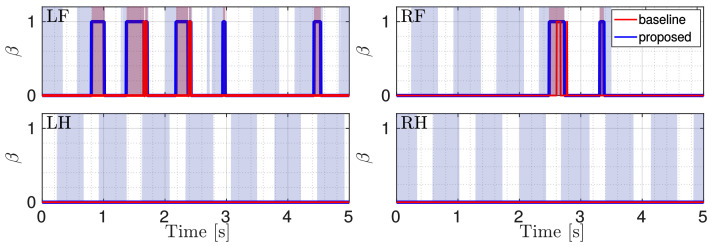
Comparison between the flags. The red one is obtained with the baseline approach, the blue one with the proposed approach. The gray shaded area shows stance phases and the red shaded area indicate the ground truth.

Figure 9 shows $\bar{\Delta V}$ and ΔP when the robot trots onto ice slabs. The shaded red areas indicate the actual slipping. Figure 10 shows the comparison between the flags obtained using the baseline and the proposed approaches. We see that the slip detection of correctly identified slipping stance legs does not affect the slip detection of the other stance legs (their flags continue to be 0 thanks to the fact that ΔP remains below $\epsilon_{p}$). Additionally, in this simulation, the proposed approach is more efficient in detecting the slippage of each foot for its entire duration.

## 5. Experimental Results

In this section, we show experimental results on HyQ. A first experiment was done with the robot walking on non-slippery terrain. A second experiment was performed using a slippery patch for the robot to walk on. To make the patch slippery, we sprinkled its surface with soap. For the experiments we used only crawl as gait. We recorded data from these two experiments and then we used them offline to carry out a proper tuning procedure of the thresholds. We found the minimum $\epsilon_{p}$ and $\epsilon_{v}$ that do not detect any slipping in the first experiment, and 95% of the actual slips in the second one.

After the first experiment we chose the following parameters: $\epsilon_{p}=0.04$, m=0.3, $\epsilon_{v}=percentile\left(\bar{\Delta V},95\%\right)$. During the test, LH and RF slipped at the same time, so, for the comparison, we implemented the *multiple leg slip detection* method of the baseline. We set $\epsilon_{v_{BL}}=1$ as threshold for the baseline approach.

**Figure 11:**
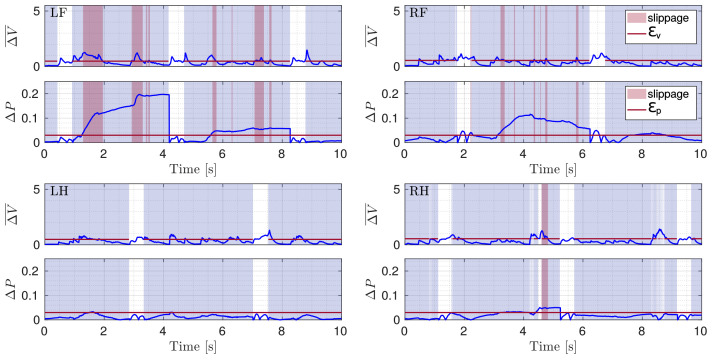
Plots of $\bar{\Delta V}$ and ΔP (blue) with respective thresholds (red line) illustrated for the four legs (LF, RF, LH, RH) during a crawl gait. The gray shaded area shows stance phases and the red shaded area marks slippage.

**Figure 12:**
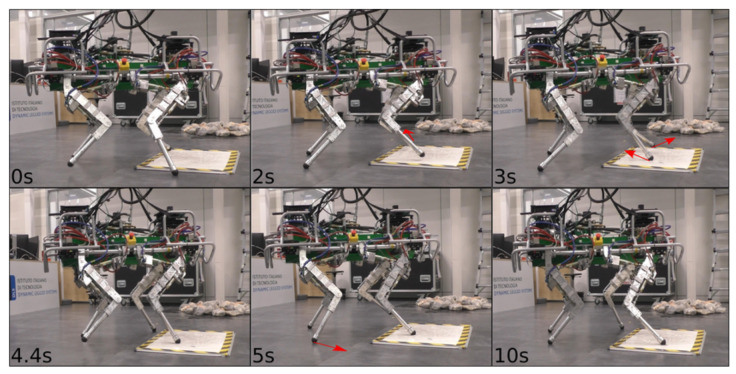
Experiment of the HyQ robot traversing slippery terrain patches with a crawl gait (low friction patches obtained by sprinkling a whiteboard with soap). The image sequence starts from the top left to right and continues at the bottom left. The red arrows indicate slipping feet.

**Figure 13:**
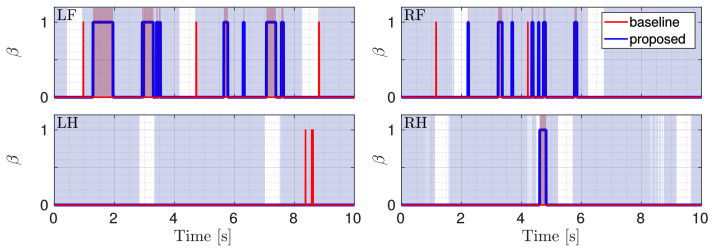
Comparison between the flags. The red one is obtained with the baseline approach, the blue one with the proposed approach. The gray shaded area shows stance phases and the red shaded area indicate the ground truth.

Although only the LH and RF legs were placed in slipping conditions, during the experiment, also for the RH leg a slippage occurred. LH was the only non-slipping leg (the reader can refer to the attached video). Figure 11 shows $\bar{\Delta V}$ and ΔP for all the four legs during the slippery terrain experiment shown in Figure 12. Figure 13 shows the comparison between the flags obtained using the two methods.

As can be seen in Figure 11 and Figure 13, all the detections are correctly performed. Additionally, in the experiments, the results obtained with the proposed method are better, as they detect the actual slipping events, there are no false positives and the flags are also able to show the duration of the slipping. There are, instead, false positives in the baseline approach, for which a slippage is detected for LH although it did not actually slip.

## 6. Conclusions and Future Works

In this paper, we presented a novel slip detection approach for legged robots based on kinematics, which makes use of velocity and position measurements at the ground contacts. In the field of legged robots, a kinematic-based approach is more suitable than a force-based approach, which involves the use of 6-axis force/torque sensors at the feet. The provided method shows that it is possible to detect a slippage quickly and effectively relying on the foot positions and velocities expressed in the base frame. This allows avoiding problems related to drift, which usually happen when using the world frame. We proposed a method suitable for different types of locomotion and which is applicable to situations where the robot is required to change its velocity. Then we proved the effectiveness of the algorithm through the results obtained in simulation tests and in experiments. We also compared these results with those obtained using an already existing algorithm, showing that our implementation is more robust.

Future work includes the analysis of the maximum amount of slippage tolerable in the context of locomotion, that can preserve stability. Furthermore, we plan to implement an estimation of the friction properties of the terrain during the locomotion. This can be useful to set different levels of “cautiousness ”, selecting more or less conservative gaits according to the situation. In the future we can fuse the proposed approach with information coming from vision, that could provide a default value for the friction coefficient together with an estimate of its roughness. Then we can move on to the implementation of a recovery strategy, that is essential for locomotion on very slippery terrain such as ice and in situations where the inclination of the terrain is wrongly estimated.

## Data Availability

The data that supports the finding of this paper is available from the corresponding author upon reasonable request.

## Abbreviations

The following abbreviations are used in this manuscript:

DLS	Dynamic Legged Systems
IIT	Istituto Italiano di Tecnologia
ROS	Robot Operating System
HyQ	Hydraulically actuated Quadruped
PTAL	Proprioceptive Terrain-Aware Locomotion
GRF	Ground Reaction Force
IMU	Inertial Measurement Unit
UKF	Unscented Kalman Filter
DoF	Degree of Freedom
HAA	Hip Adduction-Abduction
HFE	Hip Flexion-Extension
KFE	Knee Flexion-Extension
RCF	Reactive Controller Framework
LF	Left-Front
RF	Rigth-Front
LH	Left-Hind
RH	Right-Hind
